# Changes in postural sway and cortical activities after napping

**DOI:** 10.1371/journal.pone.0320926

**Published:** 2025-04-09

**Authors:** Hui-Ya Chen, Li-Yuan Chen, Shu-Zon Lou, Chun-Ling Lin

**Affiliations:** 1 Department of Adapted Physical Education, National Taiwan Sport University, Taoyuan, Taiwan; 2 Department of Physical Therapy, Chung Shan Medical University, Taichung, Taiwan; 3 Physical Therapy Room, Chung Shan Medical University Hospital, Taichung, Taiwan; 4 Department of Occupational Therapy, Chung Shan Medical University, Taichung, Taiwan; 5 Department of Electronic Engineering, National Taipei University of Technology, Taipei, Taiwan; National University of Sciences and Technology, PAKISTAN

## Abstract

Lowered arousal state after napping may lead to poorer standing balance and the need to recalibrate the sensory organization system. This study aimed to examine the changes in postural sway and sensory associated cortical activities immediately after waking from a nap. A convenience sample of young adults (7 males and 5 females, 21.0 ± 2.3 yr.) was recruited. Before and after a 50-min lying-down nap, participants were asked to stand quietly with eyes open/closed on a firm/foam surface, and electroencephalography (EEG) in theta, alpha, beta, and gamma bands in sensory association areas was recorded. All participants self-reported that they fell asleep during the 50-min period provisioned for nap (Karolinska Napiness Scale before nap 4.2 ± 1.1, after nap 5.7 ± 0.8). The average time taken to finish data collection after waking the participants was 19.0 ± 4.0 minutes. The results showed less postural sway (t_11_ =  2.726, p =  0.02) and increased frequency of postural sway (t_11_ =  -3.339, p =  0.007) after nap in the eyes-open firm-surface condition. The EEG results revealed decreased activity in the alpha (F_1,9_ =  15.540, p =  0.003) and gamma (F_1,9_ =  6.626, p =  0.030) bands in the right parietal area after nap, and increased beta power in the left occipital area (Z =  -2.241, p =  0.025). In conclusion, after waking from a nap, healthy adults show increased changes in direction of postural sway which is effective in decreasing postural sway in eyes-open firm-surface condition. Even in healthy adults without worsen postural performance after nap, the EEG results suggested a decrease of efficacy in dealing with sensory challenges within twenty minutes post napping. This study contributes to the understanding of the mechanisms underlying changes in balance control after napping, which might help fall prevention programs for the elderly.

## Introduction

The time of day when the nap is taken influences alertness and postural performance. Previous research indicates that the increased sleepiness experienced from 1:30–4 pm increases postural sway [1, 2] and the risk of accidents [3]. The possible mechanisms underlying poorer standing balance immediately after waking from a nap include a lowered arousal state and the need to recalibrate the sensory system.

Visual, vestibular and somatosensory information are the three sensory modalities essential for postural balance. High-level processing and integrating these three modalities is termed sensory organization [4]. During sensory organization, relative weights are given to these three sensory modalities depending on contexts [5]. For instance, when standing in the dark, the relative weight of visual input decreases and that of somatosensory and vestibular information increases. Apart from sensory deprivation, there are contexts involving sensory conflicts. For instance, when we stand on a compliant surface, somatosensory input is misleading and conflicting with inputs from the other two sensory modalities such that the use of somatosensory input has to be inhibited. It is likely that after a period of nap, the processing of sensory organization in the central nervous system needs to be recalibrated such that the best relative weights of the three sensory inputs are finely adjusted.

Electroencephalography (EEG) recordings may help to reveal the mechanisms underlying changes in balance control after nap. Higher-level sensory organization occurs in sensory association cortices, such as the parietal, temporal, and occipital lobes, which links perception to action and involves interplay between cognitive and perceptual processing [4]. Our previous EEG study exploring continuous balance maintenance documented that eye closure is associated with increased alpha and beta activities in all sensory association areas, which may be linked to the neural process of shifting relative weight from the visual modality to the other two modalities [6]. We also found that eye closure is associated with increased fast beta activity in the bilateral parietal-temporal-occipital areas, which represents neural processes of high-level sensory organization with greater cognitive loads [6]. Furthermore, compared to the effects of eye closure on EEG activity, misleading somatosensory information effects are of smaller amplitude and in a different direction. Decreased alpha activity in left parietal-temporal-occipital areas and decreased beta and fast beta activities in bilateral parietal-temporal-occipital areas may reflect the processing of vestibular information [6]. However, our literature review failed to find any research on the influences of taking a nap on the sensory organization mechanisms of standing balance.

This research aimed to explore the mechanism controlling standing balance immediately after waking from a nap by examining sensory organization ability and EEG cortical activities. Whole-scalp cortical activities in the theta, alpha, beta, and gamma EEG bands in four sensory conditions, with sensory difficulty increasing in a stepwise manner, were examined. We hypothesized worsen postural performance in healthy young adults during sensory challenging conditions after nap. We also hypothesized that even in healthy young adults, EEG in sensory association areas would show changes after nap.

## Materials and methods

### Participants

A convenience sample of twelve young adults (7 males and 5 females, 21.0 ± 2.3 yr.) was recruited during May and June 2018. All participants were right-handed and right-footed. The exclusion criteria were muscular-skeletal trauma in the past three months, current medications that may affect balance, hearing difficulty, acute or chronic ear infections, and a history of vertigo. The protocol was approved by the Institutional Review Board of Chung Shan Medical University Hospital (CS17001). All participants provided written informed consent. All methods were performed in accordance with the Helsinki Declaration. The authors had access to information that could identify individual participants during and after data collection.

### Equipment

The modified Clinical Test for Sensory Interaction in Balance [7] involved four conditions with increased sensory difficulty in maintaining balance in a stepwise manner. The visual conditions included eyes-open and eyes-closed conditions. The support conditions included standing on firm or foam surfaces, in which an Airex compliant medium-density foam (50 cm X 41 cm X 6 cm) was used. One wireless triaxial body-worn Opal inertial sensor (APDM Inc., Portland, OR, USA) was placed at L-5 with an elastic belt. The system included a set of Opals with a docking station and an access point for wireless data transmission at 128 Hz, from which the anteroposterior and mediolateral directions of change were used to quantify postural sway after wireless transmission to a laptop using Mobility Lab software from APDM.

During the modified Clinical Test for Sensory Interaction in Balance, continuous EEG was collected using a BR32S (Brain Rhythm, Taiwan), which has 32 active electrodes mounted in elastic BioSemi headcaps with electrode positions according to the extended 10–20 international system. Bluetooth wireless transmission collected data at 250 Hz. To synchronize the APDM and EEG collection, RS232 putty software was used to create another channel signal for EEG data; that is, every time the experimenter started the trial, he/she simultaneously pressed a key on the RS232 putty, and a signal was created and input into the EEG data.

### Procedure

**Fig 1:**
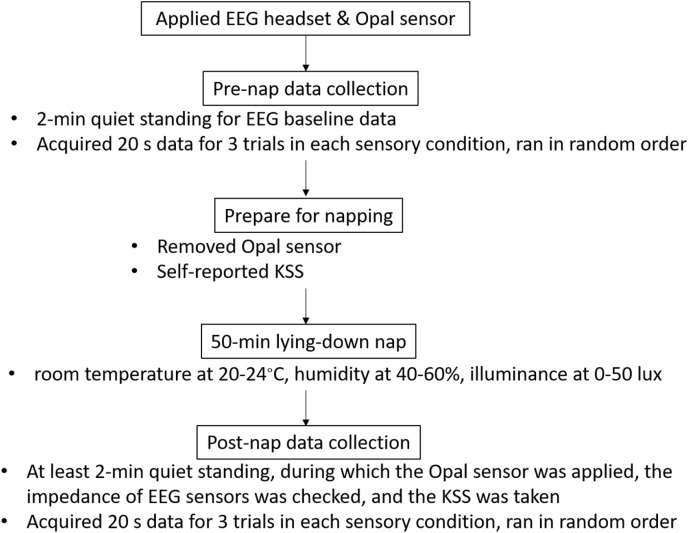
Experiment procedures. There were two data-collection sessions, separated by a 50-min lying-down nap. Before the session of pre-nap data collection, electroencephalography (EEG) and a triaxial body-worn Opal inertial sensor were applied. Each data collecting session consisted of three trials in each of the four sensory conditions, ran in random order. After the session of pre-nap data collection, the Opal sensor was removed and the 50-min lying-down nap began. Before and immediately after napping, the arousal state as self-reported by the Karolinska Napiness Scale (KSS) was taken. After napping, participants soon resumed an upright stance for at least 2 min, during which the experimenter applied the Opal sensor and adjusted the EEG sensors. Afterwards, the post-nap data collection session started as soon as possible.

The participants were told to wash their hair the day before the experiment and to avoid drinking caffeine or alcohol within 24 h. They were also instructed to wear comfortable clothes. There were two data-collection sessions, separated by a 50-min nap (Fig 1). Each session consisted of three trials in each of the four sensory conditions. To prevent frequent stepping up and down the foam surface, the two firm-surface conditions were grouped, as were the two foam-surface conditions, creating eight possible orders, which were randomly assigned.

For each session, after being fit with the EEG system and the Opal sensor, the participants stood quietly with barefoot and a comfortable stance for two minutes to prevent the postmovement beta wave [8, 9], served as baseline EEG data. The stance width was marked and made consistent throughout the experiment. When the participants reported that they were ready, the EEG started collecting data for approximately 3 s, and then the trial began and lasted for 20 s. The participants were instructed to maintain standing balance while staying relaxed, to look straight ahead. They were also told to avoid blinking.

After the pre-nap data collection session, the Opal sensor was removed, and the participants lied on a bed with a pillow and a duvet. The participants were asked to self-report their arousal state on a nine-point scale, the Karolinska Napiness Scale [10], from extremely alert (one point) to very napy (nine points). The experiment was scheduled at 1:30 pm so that the 50-min nap occurred from approximately 2 pm to 3 pm. We controlled the room temperature at 20–24°C, humidity at 40–60%, and illuminance at 0–50 lux. After 50 min, the experimenter heightened the illuminance to 500–800 lux and played soft music before orally waking the participant. The participants soon resumed an upright stance for at least 2 min, during which the experimenter finished the following procedures as soon as possible: applied the Opal sensor, adjusted the EEG sensors, checked the impedance, and took the KSS. Afterwards, the post-nap data collection session started as soon as possible, exactly as in the pre-nap data collection session.

### Data analysis

Postural sway metrics were quantified during each stance condition by Mobility Lab software. From the automatic metrics, the postural sway velocity (m/s) and frequency of the centroid postural sway (Hz) in the transverse plane were used for analysis.

EEG analyses were conducted with EEGLAB software (v2021.0) [11]. To eliminate direct current shifts and power-line noise (50–60 Hz), a bandpass finite impulse response filter between 2–50 Hz was applied in the EEG time series. By visual inspection, nonphysiological artifacts and bad continuous EEG channels were rejected for a clean independent component analysis. The average of the bilateral mastoid electrodes was used as the recording reference. Independent component analysis was then applied to EEG signals and minimizes the statistical dependence between the components. The EEGLAB plugin ADJUST was adopted to assist in automatic EEG identification and rejection of independent components reflecting stereotypical artifacts, such as line noise, eye movements, and eye blinks by combining stereotyped artifact-specific spatial and temporal features. Once artifact-independent components were identified, ADJUST could remove them while leaving the activity due to neural sources nearly unaffected [12].

Single-trial data epochs were extracted from the artifact-corrected EEG time series within a time frame extending from 0 sec to 15 sec after the RS232 input signal. Eight epochs had technical problems with the RS232 input signal; thus, there were only 2 epochs in one sensory condition in some participants. To visualize EEG spectral dynamics, for each epoch, a short-time Fourier transform was implemented for convolution of data across 200 equally spaced time points, yielding a matrix of frequencies (2–50 Hz) ×  times (0–15 sec). Then, event-related spectral perturbations (ERSPs) were derived by converting spectral density estimates to log power. Finally, all power spectral density estimates were averaged in each sensory condition. Thus, this approach yielded four matrices of frequencies (72 steps; unit of frequency was 0.6761 Hz) ×  times (200 points; unit of time was 72 milliseconds) for 2–50 Hz for each condition for each participant. The mean signal power spectrum for each condition was obtained by averaging estimates across the time dimension in the 15-sec estimated ERSPs after the RS232 input signal.

The components across twelve individuals with similar scalp topographies and ERSPs were grouped into the same component cluster using the EEGLAB plugin STUDY [11]. First, each independent component measure was reduced into a 10-dimensional measure by principal component analysis. The principal components of ERSPs were then assigned a weight of 3, and those of scalp topographies were given a weight of 5. Finally, independent components were clustered by applying the k-means algorithm. Independent components with a distance larger than three standard deviations from the mean of any cluster centroid were removed and not analyzed. Since not all the participants had the same scalp topographies after independent component analysis, the clustering components did not include all participants. In this study, to examine cortical activities during sensory organization, we analyzed five EEG frequency bands: 4–7 Hz (theta), 8–12 Hz (alpha), 13–21 Hz (beta), 22–30 Hz (fast beta), and 31–50 Hz (gamma) in the scalp topographies located in the sensory association areas. Changes in spectral power of a specific frequency band in each sensory condition were divided by those of the corresponding frequency band in the eyes-open condition on a firm surface before nap in the same clustering component for each participant.

Data were analyzed using the SPSS statistical package (PASW Statistics 14.0, SPSS Inc., Chicago, USA). The normality of all data was first tested using the Kolmogorov-Smirnov test. Separate repeated measures analysis of variance (ANOVA) with 2 times (before and after the nap) ×  4 sensory conditions were performed for each behavioral and EEG dataset. The Greenhouse-Geisser correction was used for the degrees of freedom when violations to sphericity occurred. When a significant main effect was found, preplanned simple contrasts, each compared to the eyes-open firm-surface condition, were performed with Bonferroni adjustments. In the case of nonnormally distributed data, nonparametric tests were used, i.e., the Friedman test for the 4 sensory conditions and the Wilcoxon signed-rank test for the 2 times (before and after the nap). When a significant effect was found in the Friedman test, post hoc analysis with Wilcoxon signed-rank tests was performed with Bonferroni adjustments. The significance level was set at p <  0.05.

## Results

### Behavioural data

All participants self-reported that they fell asleep during the 50-min period provisioned for nap. The average Karolinska Napiness Scale before the nap was 4.2 ± 1.1 (range 3–6), and after the nap, it increased to 5.7 ± 0.8 (range 4–7). The average time taken to finish data collection after waking the participants was 19.0 ± 4.0 minutes (range 13–25 minutes).

**Fig 2:**
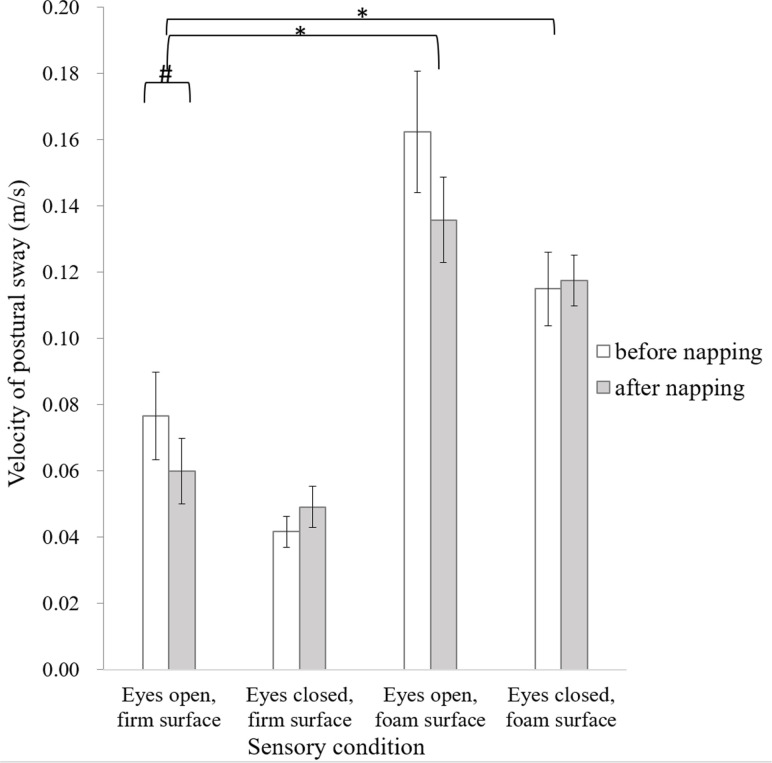
Velocity of postural sway in the four sensory conditions before and after napping. Error bars of one standard error are shown. The asterisk *  indicates significant difference (p <  0.05), whereas the hashtag # indicates marginal significance.

In terms of the velocity of postural sway (Fig 2), ANOVA revealed a main effect of condition (F_3,33_ =  33.748, p <  0.001, ηp2 =  0.754), and preplanned simple contrasts showed larger sway in the eyes-open foam-surface condition (F_1,11_ =  66.297, p <  0.001, ηp2 =  0.858) and the eyes-closed foam-surface condition (F_1,11_ =  12.978, p =  0.004, ηp2 =  0.541) than in the eyes-open firm-surface condition. ANOVA also revealed marginal significant interaction effect of condition by time (F_3,33_ =  2.866, p =  0.051, ηp2 =  0.207). The velocity of postural sway decreased after napping, at a marginal significant level, only in the eyes-open firm-surface condition (t_11_ =  2.726, p =  0.02).

**Fig 3:**
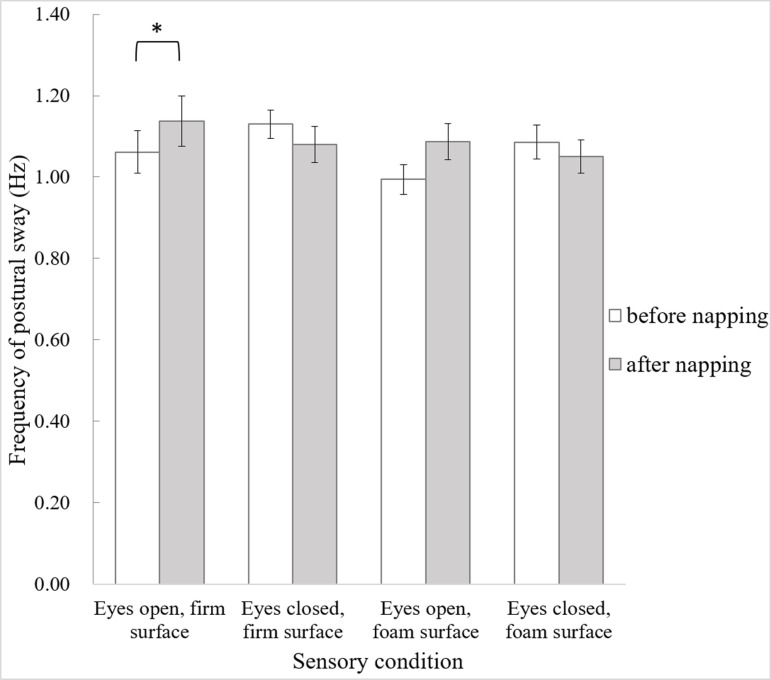
Frequency of postural sway in the four sensory conditions before and after napping. Error bars of one standard error are shown. The asterisk *  indicates significant difference (p <  0.05).

In terms of the frequency of postural sway (Fig 3), no main effects were found, but a significant interaction effect of condition by time was observed (F_3,33_ =  5.031, p =  0.006, ηp2 =  0.314). The frequency of postural sway increased after napping only in the eyes-open firm-surface condition (t_11_ =  -3.339, p =  0.007).

### EEG data

**Fig 4:**
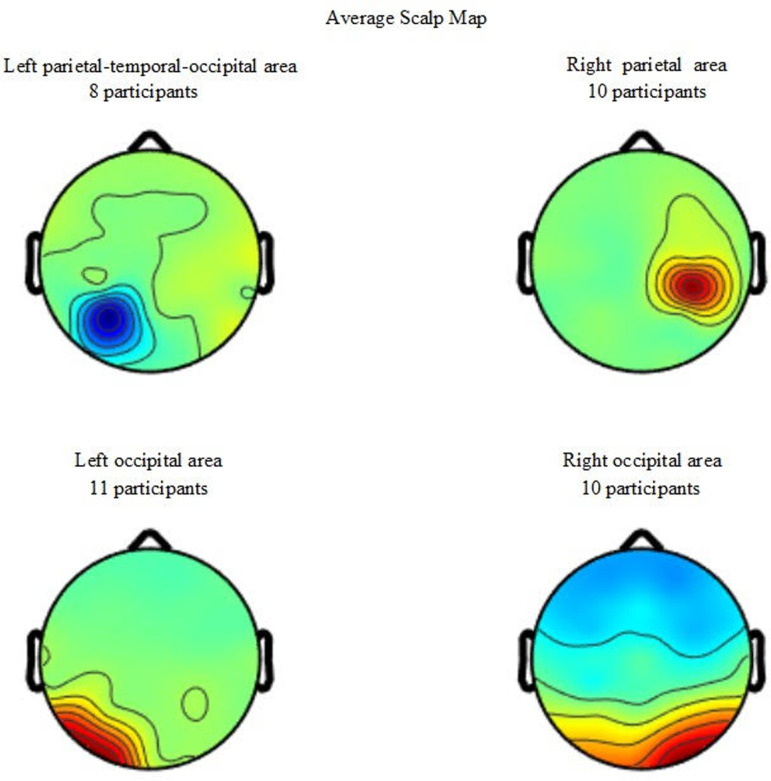
Four component clusters grouped by scalp topography. The number is the summed number of participants for that particular cluster.

**Fig 5:**
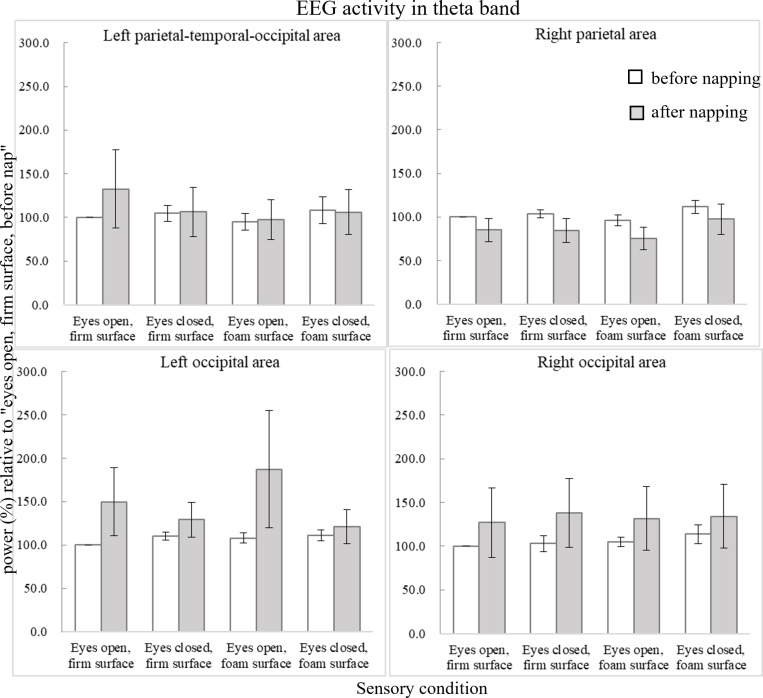
Average EEG activity (power in %) relative to the condition of “eyes open, firm surface, before nap” in the theta band (4-7 Hz). Error bars of one standard error are shown. The asterisk *  indicates significant difference (p <  0.05).

Fig 4 illustrates the average scalp maps for the four component clusters in sensory association areas. Fig 5 shows the power changes in the theta band. In the right parietal area, the main effect of sensory condition is significant (F_3,27_ =  6.058, p =  0.003, ηp2 =  0.402), showing the trend of increasing theta frequency band power with progression in postural task difficulty. However, preplanned simple contrasts reveal no significant difference between sensory conditions (all p >  0.05).

**Fig 6:**
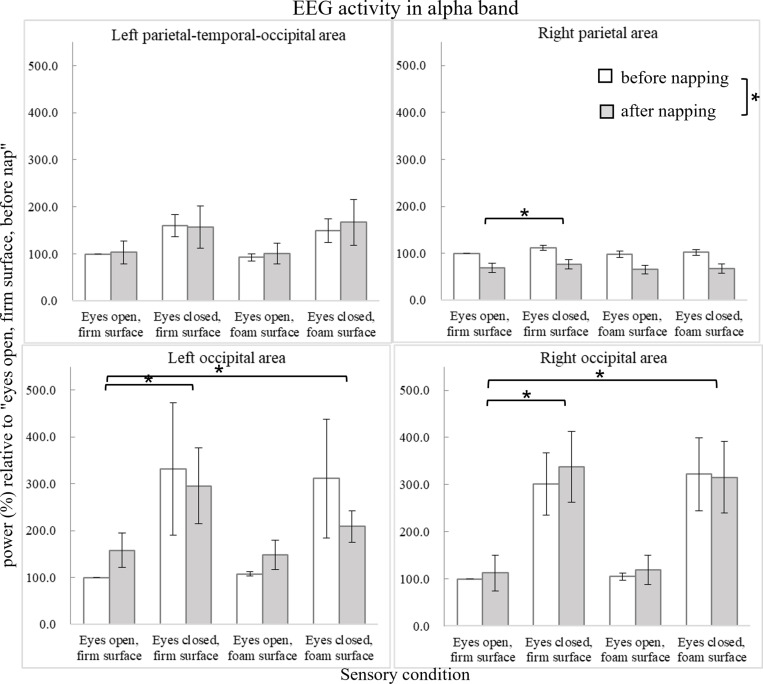
Average EEG activity (power in %) relative to the condition of “eyes open, firm surface, before nap” in the alpha band (8-12 Hz). Error bars of one standard error are shown. The asterisk *  indicates significant difference (p <  0.05).

The power changes in the alpha band as a function of condition and time are shown in Fig 6. In the right occipital area, the Friedman test revealed a significant difference between sensory conditions (X^2^(3) = 43.380, p <  0.001). Post hoc analysis revealed significantly increased power in the eyes-closed firm-surface condition (Z =  -3.845, p <  0.001) and the eyes-closed foam-surface condition (Z =  -3.845, p <  0.001) than in the eyes-open firm-surface condition. In the left occipital area, the Friedman test revealed a significant difference between sensory conditions (X^2^(3) =  33.764, p <  0.001). Post hoc analysis revealed significantly increased power in the eyes-closed firm-surface condition (Z =  -3.945, p <  0.001) and the eyes-closed foam-surface condition (Z =  -3.717, p <  0.001) than in the eyes-open firm-surface condition. In the right parietal area, ANOVA revealed a main effect of sensory condition (F_3,27_ =  3.088, p =  0.044, ηp2 =  0.255). Preplanned simple contrasts revealed significantly increased power in the eyes-closed firm-surface condition (F_1,9_ =  11.707, p =  0.008, ηp2 =  0.565) than in the eyes-open firm-surface condition. Furthermore, ANOVA revealed a main effect of time in the right parietal area (F_1,9_ =  15.540, p =  0.003, ηp2 =  0.633), showing decreased alpha power after nap.

**Fig 7:**
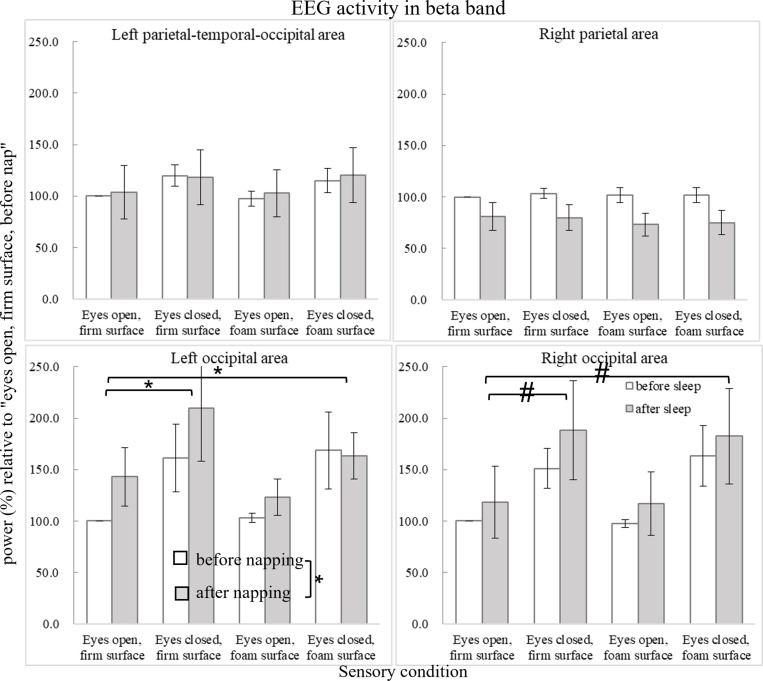
Average EEG activity (power in %) relative to the condition of “eyes open, firm surface, before nap” in the beta band (13-21 Hz). Error bars of one standard error are shown. The asterisk *  indicates significant difference (p <  0.05), whereas the hashtag # indicates marginal significance.

Fig 7 shows the power changes in the beta band. In the right occipital area, ANOVA revealed a main effect of sensory condition (F_3,27_ =  6.944, p =  0.026, ηp2 =  0.436). Preplanned simple contrasts revealed nonsignificantly increased power compared to that in the eyes-open firm-surface condition, in the eyes-closed firm-surface condition (F_1,9_ =  7.325, p =  0.024, ηp2 =  0.449) and in the eyes-closed foam-surface condition (F_1,9_ =  6.467, p =  0.032, ηp2 =  0.418). In the left occipital area, the Friedman test revealed a significant difference between sensory conditions (X^2^(3) =  14.564, p =  0.002). Post hoc analysis revealed significantly increased power in the eyes-closed firm-surface condition (Z =  -3.393, p =  0.001) and eyes-closed foam-surface condition (Z =  -2.451, p =  0.014) than in the eyes-open firm-surface condition. Furthermore, the Wilcoxon signed-rank test revealed significantly increased beta power (Z =  -2.241, p =  0.025) in the left occipital area after nap.

**Fig 8:**
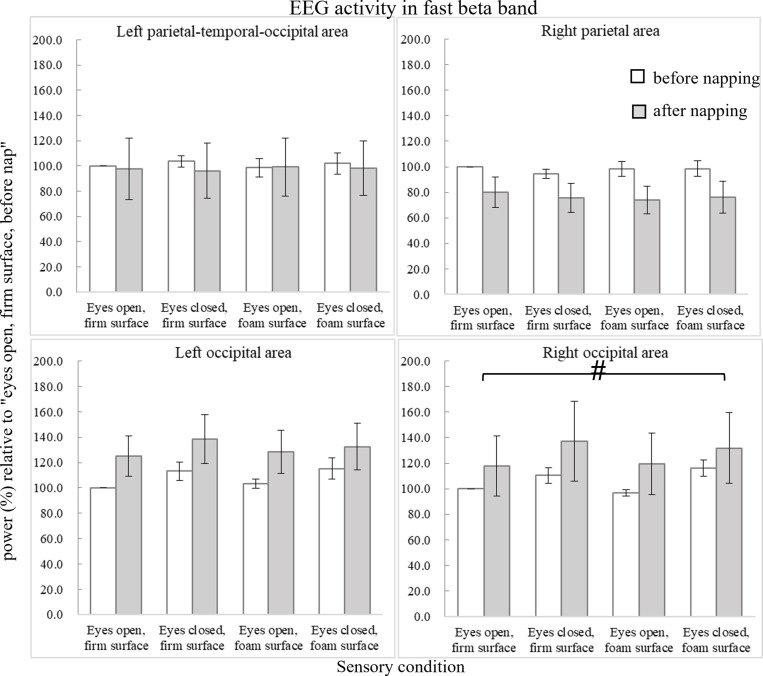
Average EEG activity (power in %) relative to the condition of “eyes open, firm surface, before nap” in the fast beta band (22-30 Hz). Error bars of one standard error are shown. The hashtag # indicates marginal significance.

Fig 8 shows the power changes in the fast beta band. In the right occipital area, ANOVA revealed a main effect of sensory condition (F_3,27_ =  4.681, p =  0.038, ηp2 =  0.342). Preplanned simple contrasts revealed nonsignificantly increased power in the eyes-closed foam-surface condition (F_1,9_ =  5.267, p =  0.047, ηp2 =  0.369) compared to that in the eyes-open firm-surface condition.

**Fig 9:**
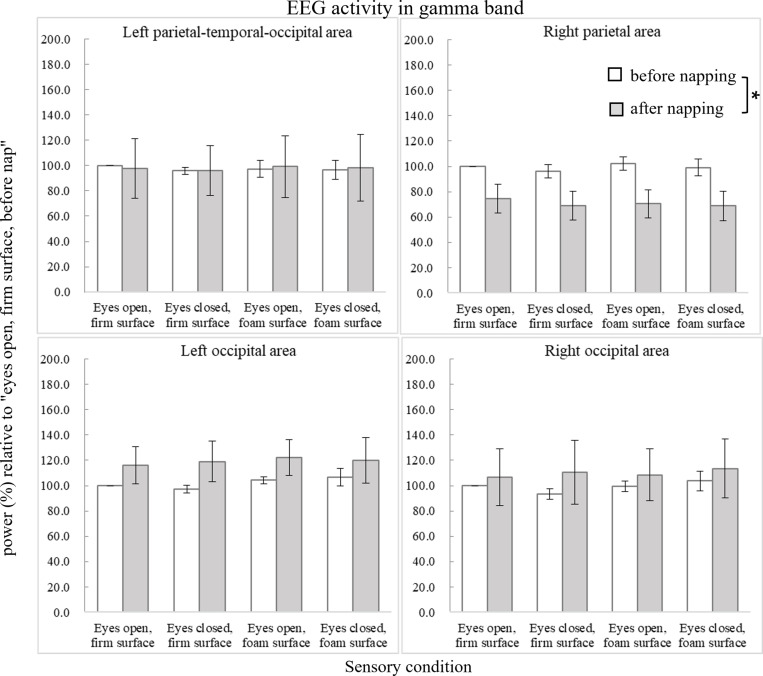
Average EEG activity (power in %) relative to the condition of “eyes open, firm surface, before nap” in the gamma band (31-50 Hz). Error bars of one standard error are shown. The asterisk *  indicates significant difference (p <  0.05).

Fig 9 shows the power changes in the gamma band. In the right parietal area, ANOVA revealed a main effect of time (F_1,9_ =  6.626, p =  0.030, ηp2 =  0.424), showing decreased gamma power after nap.

## Discussion

The behavioral results showed larger postural sway in the foam conditions, which is in line with the results of previous research [7,13]. In contrast to our hypothesis, our young healthy participants did not show worsen postural performance after napping in sensory challenging conditions, probably because their alertness only slightly decreased. The postural sway decreased after nap, accompanied by an increased frequency of postural sway in the eyes-open firm-surface condition. The frequency of postural sway of our participants was within the higher components (≧0.4 Hz), reflecting reactive postural responses to correct for postural error [14, 15]. The combined results of decreased velocity of postural sway and increased changes in direction of postural sway may indicate the postural strategy adopted by our young healthy participants after nap, in response to the increased postural errors. The strategy of higher-frequency excursions of postural sway has been found in some older adults to enhance sensory information [16], and in healthy adults during dual-tasking conditions [17]. Hence, it seems highly likely that napping heightened the challenging level of maintaining standing balance, mimicking dual-tasking or ageing effect, and caused the deliberate shift in postural strategy. However, even in our healthy young participants, this strategy of increasing postural frequency was only effective in the sensory condition with the lowest challenging level, but not in sensory conditions with eyes closure or foam surface.

Our EEG results showed increased power with eye closure. Specifically, there was increased alpha power in the bilateral occipital and right parietal areas, increased beta power in the bilateral occipital areas, and increased fast beta power in the right occipital area. Our findings of changes in EEG power due to eye closure are in accordance with the findings of Lin et al. [6] that eye closure is associated with increased alpha and beta activities in all sensory association areas and increased fast beta band activity in the bilateral parietal-temporal-occipital area. Previous research has documented that the alpha band is profoundly involved in sensory processing [18], especially in the occipital lobe [19]. Furthermore, research examining the effects of virtual reality has reported increased alpha activity while processing visual challenges during standing balance maintenance [20]. Furthermore, Chang et al. [20] documented an increased beta band in the parietal-occipital region during virtual reality, which was associated with high-level cortical modulation and sensorimotor integration. Our EEG results also showed a trend of increasing theta power with progression in postural task difficulty. In line with this finding, Hülsdünker et al. [21, 22] have reported increased theta power in fronto-central and centro-parietal regions as a function of balance task difficulty. However, our preplanned simple contrasts reveal no significant difference between sensory conditions; similar to the reports of Edwards et al. [23]. The contrary findings might be explained by the predictability of postural perturbation [24], as Hülsdünker et al. [21, 22] have introduced oscillations of supporting surfaces, thus creating more unpredictability.

Our EEG results also showed decreased alpha and gamma power in the right parietal area after nap compared to before nap. All of our participants were right-handed and right-footed, and the parietal area of the nondominant right hemisphere is known to be more important for sensory aspects of balance maintenance [23,25]. Suppressed alpha activities over the sensorimotor cortex during active movement are believed to be linked to the active state of the sensorimotor cortex [26, 27]. Thus, our results of decreased alpha band activity in the parietal area after nap may indicate the effort of the participants to maintain standing balance, in accordance with our behavioral data showing increased postural sway frequency in the eyes-open firm condition after nap. Oscillation in the gamma band is related to selective attention mechanisms in tasks with heightened attention to the environment and external visual information [28]. Ozdemir et al. described increased gamma oscillations in the elderly over sensorimotor and parietal cortices during standing balance maintenance with misleading somatosensory information, which indicates increased allocation of attentional sources to postural tasks [29, 30]. Thus, our results of decreased gamma activity in the parietal area may indicate inefficient attention attributed to the postural task after nap.

Our EEG results also showed significantly increased beta power in the left occipital area after nap. An increase in beta band activity in occipital areas has been found during postural tasks with extra cognitive loads [20,28]. Moreover, our previous research examining the effects of age on sensory organization tasks found that, compared to young adults, elderly adults have significantly increased fast beta activity in the left temporal-occipital and bilateral occipital areas in all sensory conditions [6]. The increase in fast beta activity due to aging is suggested to reflect the sustained effort that elderly adults make in all challenging sensory conditions [6]. Thus, our results of increased beta power in the left occipital area after nap may suggest greater cognitive loads to maintain standing balance after nap.

While our study provided novel findings regarding nap-induced changes in cortical correlates during challenging sensory tasks, there were some limitations. First, our sensory test had four conditions. To reduce type I statistical errors, preplanned simple contrasts with Bonferroni adjustments were performed. The need for multiple comparison correction might have compromised statistical power. Second, the laboratory setup made it difficult for the participants to completely relax and we did not have EEG checked during the nap period. Although all participants self-reported that they fell asleep during the experiment, their arousal state decreased only slightly after nap, and in some participants, the KSS score remained the same. Even though our EEG records still showed some promising results, the abovementioned facts together might have lowered the sensitivity of our research to detect early changes immediately after participants awakened from a nap. Future research could consider acquiring high-density EEG data to provide additional insights into the spatial localization of functional brain areas. Compared to conventional EEG, high-density EEG provides better source localization and reduces volume conduction effects [31], allowing more precise mapping of neural oscillatory changes [32]. Furthermore, its finer temporal resolution supports time-frequency and connectivity analyses to examine cortical interactions [33].

## Conclusions

In the first 20 minutes after waking from a nap, healthy young adults show slightly decreased alertness and decreased postural sway through the strategy of increasing changes in direction of postural sway in eyes-open firm-surface condition. This suggests the deliberate effort to decrease postural sway after nap, which is only effective in the sensory condition with the lowest challenging level. Even in healthy adults without worsen postural performance after nap, the EEG results reveal decreased cortical activities in the alpha and gamma bands in the right parietal area, suggesting increased effort made by participants but inefficient attention attributed to the postural task. The EEG also shows increased beta power in the left occipital area after nap, suggesting greater cognitive loads to maintain standing balance after nap. The changes in EEG power after napping are not related to specific sensory conditions, i.e., no interaction effect with sensory tasks, but suggest a decrease of efficacy in dealing with sensory challenges in standing balance tasks after nap.

This study contributes to the mechanisms underlying changes in balance control within twenty minutes post napping, which might help fall prevention programs for the elderly. Even in healthy young adults without obvious decrement in postural performance after napping, the postural control system is characterized with inefficient attention and greater cognitive loads. A more obvious trend could be anticipated in the elderly population; thus allowing more time between waking and resuming standing posture could be incorporated in the fall-prevention health education. In addition, strategies to recover attention and to reallocate cognitive resources could be developed.

## Supporting information

S1 Data All relevant data analysed in this study. (XLSX)
